# Is IgE or eosinophils the key player in allergic asthma pathogenesis? Are we asking the right question?

**DOI:** 10.1186/s12931-018-0813-0

**Published:** 2018-06-08

**Authors:** Andrea Matucci, Alessandra Vultaggio, Enrico Maggi, Ismail Kasujee

**Affiliations:** 1 Immunoallergology Unit, Azienda Ospedaliero-Universitaria Careggi, Largo Brambilla 3, 50134 Florence, Italy; 2 Center for Research, Transfer and High Education DENOTHE, University of Florence, Florence, Italy; 3 Novartis Pharma AG, Basel, Switzerland

**Keywords:** Immunoglobulin E, IgE, Eosinophil, Interleukin 5, IL-5, Asthma pathogenesis; anti-IgE, Anti-IL-5

## Abstract

Bronchial asthma (BA) is a chronic inflammatory disease with a marked heterogeneity in pathophysiology and etiology. The heterogeneity of BA may be related to the inducing mechanism(s) (allergic vs non-allergic), the histopathological background (eosinophilic vs non-eosinophilic), and the clinical manifestations, particularly in terms of severity and frequency of exacerbations. Asthma can be divided into at least two different endotypes based on the degree of Th2 inflammation (T2 ‘high’ and T2 ‘low’). For patients with severe uncontrolled asthma, monoclonal antibodies (mAbs) against immunoglobulin E (IgE) or interleukin (IL)-5 are now available as add-on treatments. Treatment decisions for individual patients should consider the biological background in terms of the “driving mechanisms” of inflammation as this should predict the patients’ likely responses to treatment. The question is not whether an anti-IgE or an anti-eosinophilic strategy is more effective, but rather what the mechanism is at the origin of the airway. While IgE is involved early in the inflammatory cascade and can be considered as a cause of allergic asthma, eosinophilia can be considered a consequence of the whole process. This article discusses the different roles of the IgE and IL-5/eosinophil pathways in the pathogenic mechanisms of airway inflammation occurring in allergic asthma, and the possible reasons to choose an anti-IgE mAb or anti-IL-5 treatment.

## Background

Bronchial asthma (BA) is a chronic inflammatory disease with a marked heterogeneity in etiology, pathophysiology and clinical aspects, leading to the identification of different phenotypes (early onset atopic/allergic, eosinophilic, exercise induced, obesity and paucigranulocitic) [1] Subsequently, taking into account the molecular mechanisms underlying the pathophysiology of bronchial inflammation, the concept of endotypes has been introduced [2]. BA is often sustained by allergic sensitisation, which leads to bronchial hyper-responsiveness and acute bronchoconstriction in response to specific and non-specific triggers. For many years, from a pathogenic perspective, the focus of research has been on the role of T cells in the initiation and perpetuation of inflammation [3, 4]. In particular, T helper 2 (Th2) cells have been identified as the cells involved in controlling immunoglobulin E (IgE) production because of their ability to produce interleukin (IL)-4 and IL-13, and influence the functioning of eosinophils through the actions of IL-5 [5]. The presence of Th2 cells in the bronchoalveolar lavage from atopic asthmatics was clearly demonstrated [6], However, subsequent analyses demonstrated that at least two different endotypes could be proposed based on the degree of Th2 inflammation and called T2 ‘high’ and T2 ‘low’ [6, 7]. Regarding the type of bronchial infiltrate, eosinophils have been considered important pro-inflammatory and epithelial-damaging cells, both in allergic and non-allergic asthma [8], although alternative inflammatory cells such as neutrophils may be involved [9]. Allergic (or atopic) asthma represents the most frequent endotype of asthma representing over the 60% whereas non atopic eosinophilic phenotype represents about 25–30% of the cases [10]. Approximately 5–10% of patients suffering from asthma have severe refractory asthma, among them non-eosinophilc forms are frequents [11, 12]. Particularly, based on some clinical data, 55% are eosinophilc forms, 20% are neutrophilic forms, 18% paucigranulocytic and 6% a mixed forms [13]. It is important to underline that among all severe forms of asthma, particularly in young subjects, a specific IgE sensitization is demonstrable in a significant proportion of patients [14].

The goal of asthma treatment is to achieve good control of symptoms, to reduce exacerbations and to improve quality of life. Guidelines recommend adapting the level of treatment to the level of disease severity, and this approach has been demonstrated to be effective in the majority of asthma patients overall. However, it is known that a small but significant proportion of patients do not achieve adequate control despite optimised treatment, and these patients are frequently prescribed high doses of oral steroids in an attempt to achieve control [15]. For patients with severe uncontrolled asthma, monoclonal antibodies (mAbs) against IgE or IL-5 are now available as add-on treatments to inhaled corticosteroid (ICS) plus long-acting β_2_-agonist (LABA) therapy. For Global Initiative for Asthma (GINA) Step 5 patients, a targeted therapy approach is recommended; omalizumab, a humanised mAb that selectively targets IgE, and mepolizumab and reslizumab, two additional humanised mAbs targeting IL-5, are available treatment options [16, 17]. This article discusses the different roles of the IgE and IL-5/eosinophil pathways in the pathogenic mechanisms of airway inflammation occurring in asthma, shedding light on the parts played by each in the induction and maintenance of the inflammatory process.

## The discovery of IgE and its role in allergic inflammation

While the discovery of IgE in 1966 brought to an end the search for the elusive reagin, it unlocked an era of discovery that investigated the genetics, structure, functions and clinical applications of this immunoglobulin [15]. As has been recognised since the early part of the twentieth century, IgE has unique properties among the immunoglobulin isotypes in its abilities both to induce extremely rapid pathological responses and to act as a highly sensitive immunological amplifier. Furthermore, it is well established that IgE levels are increased in patients affected by atopic conditions and that IgE provides the critical link between the antigen recognition role of the adaptive immune system and the effector functions of mast cells and basophils at mucosal and cutaneous sites of environmental exposure [18, 19]. These functions have made IgE an attractive target for pharmacological intervention with IgE blockade having clinical potential across many different therapy areas.

Despite focusing on IgE for several years, there has been relatively little consideration of pathways outside that of the mast cell and the acute phase reaction. In fact, after the discovery of IgE a great deal of attention was paid to explaining the mechanisms of acute allergic reactions such as anaphylaxis. Around the late 1970s, the role of IgE was believed to be limited to the activation of mast cells and basophils leading to the release of mediators that induced the acute phase of allergic inflammation and were responsible for the acute symptoms. From a clinical perspective, the early phase of an allergic reaction, occurring within a few minutes of exposure, is followed by the late phase, which is more complex in terms of the cellular and molecular ‘components’ involved. It is known that allergic patients sensitised to perennial allergens (or polysensitised) are constantly exposed to these substances resulting in ongoing inflammation. Observation of a time-limited early phase reaction is a rare occurrence that, in any case, develops into a late phase reaction as a consequence of the production of several mediators, cytokines and chemokines by activated mast cells and basophils [20]. In the early 1990s, the discovery of Th2 lymphocytes, their role in controlling IgE production and in the late phase of allergic inflammation, reduced the biological importance of IgE antibodies [21]. Thus, traditionally IgE antibodies were believed to be responsible for the classic “early phase” of an allergic reaction and considered to have only a minor (peripheral) role in the “late phase” reaction. During this period, IgE lost some “popularity” and was only considered of importance from a diagnostic perspective to confirm forms of allergic asthma through skin and in vitro testing. In particular, total IgE did not have any diagnostic significance. Despite interest in IgE antibodies being lessened, a number of studies achieved decidedly important results regarding not only the biological role of IgE but also the therapeutic effects of IgE-blocking mAbs [22]. This was the turning point in defining the biological role of IgE and, in 2003, the introduction of omalizumab, a humanised mAb that selectively binds to IgE, for the treatment of moderate to severe persistent allergic asthma (and later also chronic idiopathic urticaria), marked a milestone in both mAb and anti-IgE therapy. The coming decades will likely witness further advances in our understanding of IgE biology, together with the introduction of next-generation anti-IgE therapies and innovative strategies to manipulate the IgE axis and modulate allergic disease.

## What is the role of IgE in severe asthma?

The biological role of IgE is complex and related to its ability to influence the functioning of several immune and structural cells involved in the pathogenesis of chronic allergic inflammation. The biological pathways that IgE uses to influence cell activity rely on interactions with specific receptors. Two classes of receptors have been identified: high-affinity (FcεRI) receptors and low-affinity CD23 (or FcεRII) receptors. FcεRI receptors are not only expressed by mast cells and basophils but also by dendritic cells (DCs), airway smooth muscle cells (ASMCs), epithelial cells, endothelial cells, and eosinophils [23–26]. DCs can provide all of the co-stimulatory signals required for activation of T cells, which play a key role in the pathogenesis of BA. DCs are dedicated antigen-presenting cells and are key in the induction of Th2 cell activation in the primary immune response to allergens [27, 28]. The ability to present antigens is amplified by IgE bound to FCεRI receptors expressed on the surface of DCs. It has been shown that IgE captures the allergens, facilitating their presentation to memory Th2 lymphocytes [29]. FcεRI–IgE-dependent allergen presentation by DCs may critically lower the atopic individual’s threshold to mount allergen-specific T cell responses. In fact, the targeting of allergens to FcεRI via IgE leads to a 1000-fold increase in the activation of T cells in addition to the production of chemokine ligand 28 (CCL28), a chemokine that selectively attracts Th2 lymphocytes [30]. Furthermore, the activation of allergen-specific Th2 cells is associated with an amplification of allergen-specific IgE production in a vicious cycle of the pathogenic mechanisms of allergic asthma. IgE antibodies are also able to negatively modulate the innate function of plasmocytoid DCs. In these cells, the activation of FcεRI receptors blocks, or at least reduces, the intracellular signals involved in type I interferon (IFN) production [31]. The reduction in IFN production correlates with the defect in anti-viral response in allergic asthma patients [32]. Moreover, viral respiratory infections can be the initial cause of asthma, and can result in exacerbations and worsen severity [33]. Research to understand the relationship between IgE and virus infection susceptibility was spurred, in part, by the results of the clinical trial performed using omalizumab in children with asthma. The study found that anti-IgE therapy was able to reduce the exacerbations typically occurring in the spring but mainly in the fall when the children go back to school. It is known that the majority of the exacerbations are induced by viral respiratory infections [33]. The relationship beetwen virus and IgE is confirmed by the fact that respiratory syncytial virus, parainfluenza virus, Ebstain-Barr virus are able to induce a specific IgE antibody response [34–36]. Furthermore, the mechanisms by which IgE antibodies and viral infection may interact during asthma exacerbations could be related to the up-regulation of the γ chain of FcεRI receptors induced by IFN [37].

The importance of B lymphocytes to present antigens for antibody production is well documented and IgE fixed to the membrane through CD23 molecules (IgE low-affinity receptors) increases their capacity to capture allergens, amplifying the allergic response [38, 39]. In addition, some very interesting data have suggested that omalizumab may modulate human B-cell functions, including IgE synthesis [40].

The histopathological hallmark of BA consists in the constant presence, even in mild and intermittent forms of the disease, at the level of the bronchial mucosa of epithelial lesions, thickening of basement membrane, and inflammatory infiltration by activated eosinophils [41]. It has been shown that human blood eosinophils express all chains of the FcεRI receptor and can be influenced by IgE antibodies [42, 43]. Despite being fully saturated by IgE, the real function of this receptor on human eosinophils remains incompletely defined [44]. However, a direct effect of IgE on eosinophils is supported by the demonstration that omalizumab induces the apoptosis of these cells [44]. Table 1 summarises the effects of IgE on eosinophil function [42, 45–47].

**Table 1:** Direct effects of IgE on eosinophil functions

FcεRI-mediated	FcεRII-mediated
Activation and degranulation [32]	Expression of integrins (increased tissue migration) and prolonged survival [33]
Release of eosinophil peroxidase [34]	Release of TNF-α [35]

Among structural cells, airway smooth muscle cells (ASMCs) have been considered for many years to only be involved in bronchoconstriction during acute exacerbations of asthma. We need to underline that the activation of ASMCs that express FCεRIs on their surface may be, at least in part, the consequence of the involvement of the IgE pathway [24]. IgE directly activates ASMCs to produce cytokines [IL-4, IL-5, IL-13, tumour necrosis factor (TNF)-α, thymic stromal lymphopoietin (TSLP)], chemokines (CCL5, CCL11, CXCL8, CXCL10) and the traditional mediators, and cause ASMC proliferation and contraction [48]. The activation of ASMCs leads to their hypertrophy and hyperplasia, both of which correlate with asthma severity [5]. Furthermore, upon IgE-driven stimulation, ASMCs also produce and secrete extracellular matrix proteins that are key factors involved in airway wall remodelling. These effects represent the biological background that explains why anti-IgE mAbs prevent extracellular matrix and collagen deposition, and airway remodelling [49]. To definitively define the role of IgE on ASMCs, all the data must be confirmed in a not only in experimental models but also in a large series of human casistics of asthma.

The IgE biological network also includes effects on airway epithelial cells that express low-affinity IgE (CD23) receptors, which are involved in the transport of IgE-allergen complexes across the polarised airway mucosal barrier [50]. Since epithelial cells are the first cells to be exposed to inhaled allergens, they play a key role in the initiation of allergic airway inflammation, and several studies have identified an important role of the airway epithelial-derived cytokines, IL-25, IL-33, and TSLP in asthma pathogenesis [51]. Overall, the wide range of functions of IgE place it at the center of the pathogenic mechanisms of the allergic inflammatory process.

## What is the role of eosinophils in severe asthma?

Eosinophils were first identified in the late nineteenth century and eosinophilia has been known to be associated with a wide variety of conditions, including asthma and atopic diseases [52]. The relatively recent discovery of IL-5, in 1980 [53], its interaction with eosinophils, and subsequent results of anti-IL-5 blocking mAb treatment in patients with asthma confirmed the importance of IL-5 in eosinophil-mediated inflammation in humans [54, 55].

Increased numbers of eosinophils have been reported in the peripheral blood of patients with eosinophilic disorders such as asthma. Eosinophilic inflammation of the airways characterises disease severity in subsets of individuals with severe asthma and there is a direct relationship between eosinophil count and the frequency of asthma exacerbations [56–58]. Eosinophil differentiation, activation and survival mainly depends upon the effects of IL-5. This cytokine, produced at the bronchial level by Th2 cells as well as by mast cells and basophils, circulates through the blood and arrives at the bone marrow where it stimulates eosinophil progenitors, which migrate towards the bronchial walls under the effect of chemokines such as eotaxins (CCL11) [59]. However, other cytokines are also able to directly influence eosinophils. Granulocyte–macrophage colony stimulating factor (GM-CSF) and IL-3 activate and enhance eosinophil functions, such as cytotoxic killing, superoxide production, leukotriene production, and Ig-induced degranulation [60]. In addition, it should be underlined that eosinophils are greatly influenced by prostaglandin D_2_ (PGD_2_) in that they express, like Th2 cells and basophils, the specific prostaglandin D_2_ receptor 2 (DP2 or chemoattractant receptor-homologous molecule expressed on T-Helper type 2 cells [CRTH2]) [60]. Notably, mast cells, upon IgE-dependent activation, are a main source of PGD_2_ [61]. Overall, these findings indicate that the biology of eosinophils may be indirectly influenced by the IgE pathway.

Accumulation of eosinophils at the bronchial level causes damage by degranulation and release of toxic proteins such as eosinophil-derived neurotoxin, eosinophil cationic protein, eosinophil peroxidase, and major basic protein. Airway remodelling is the consequence of ongoing inflammation and repair, and there is increasing evidence that eosinophils are important in the pathophysiology of this process in both allergic and non-allergic asthma. Even though the main, and well-known, function of eosinophils relate to the induction of bronchial wall damage as final effector cells, they also represent the source of a number of regulatory and pro-inflammatory cytokines (IL-3; IL-4; IL-6; GM-CSF; TNF-α; transforming growth factor-β) and chemokines (eotaxins [CCL11]; RANTES [CCL5]) [59].

Effective antigen presentation to Th cells by human eosinophils has been recently confirmed. In fact, GM-CSF-stimulated human eosinophils can act as antigen-presenting cells to stimulate Th-cell responses against a range of antigens including allergens, an ability that may help the development of allergic disease [62].

A new category of cells, belonging to the innate immune system, has recently been identified as the source of cytokines involved in the pathogenesis of BA [63]. Among them the so-called innate lymphoid cells (ILC2) are able to produce Th2 cytokines, particularly IL-5, and these cells have been shown play a role, at least in amplyfing the bronchial inflammation [64, 65]. Unlike Th2 lymphocytes, ILC2s lack T cell receptors and are directly activated by specific cytokines, such as IL-25, IL-33, and TSLP, produced by epithelial cells after activation by allergens, viruses, and pollutants. These data have allowed a more precise distinction of hyper-eosinophilic allergic forms of asthma in which the classic Th2 cells are the director of the process in a typical adaptive immune response, from non-allergic hyper-eosinophilic asthma in which ILC2s seems to drive of the process [66].

## Relative importance of IgE compared with eosinophils in severe asthma

**Fig. 1:**
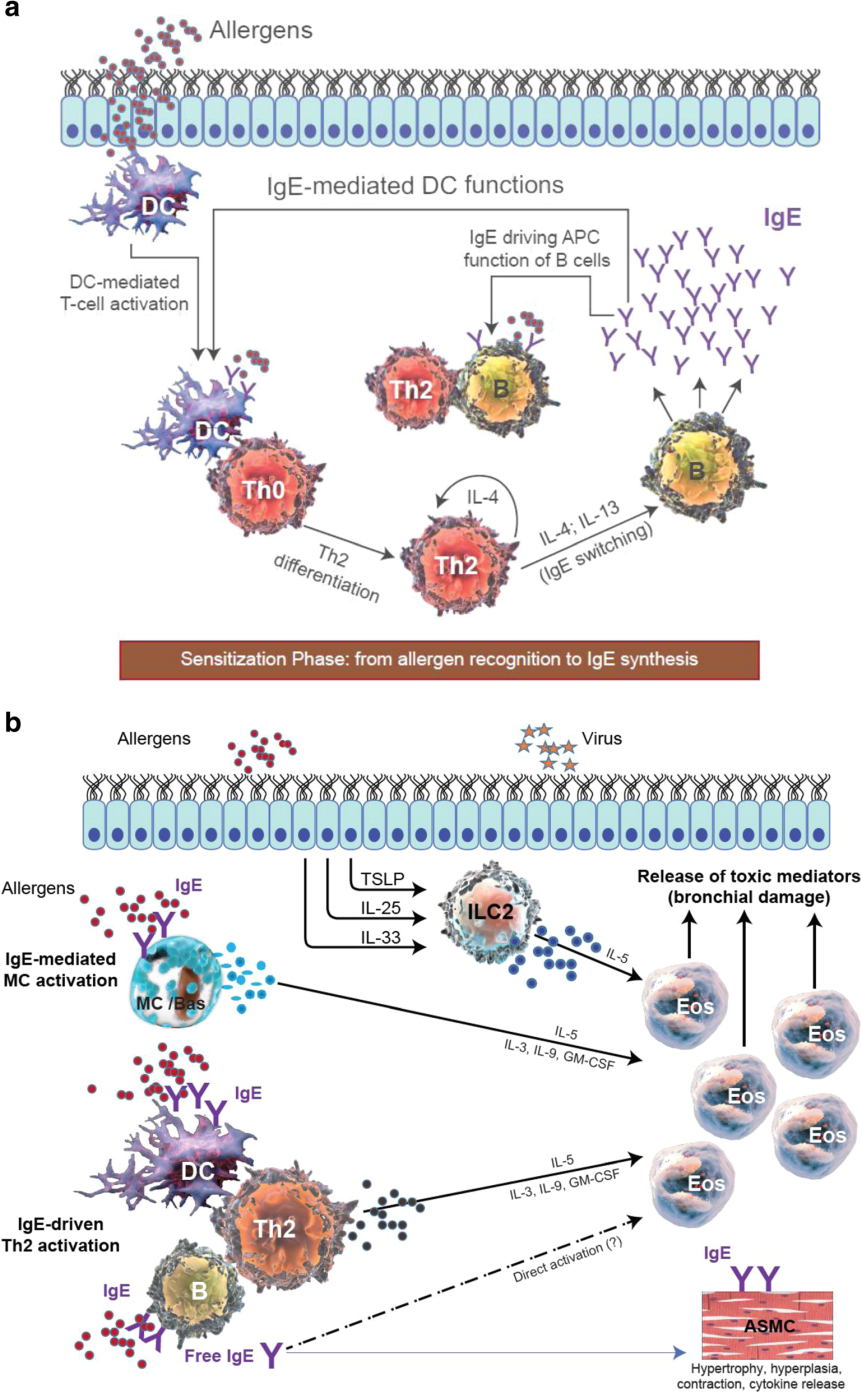
Cellular components and pathways involved in (**a**): acute (sensitisation), and (**b**): chronic (cellular damage) phases of allergic asthma pathogenesis

The availability of mAbs to treat BA appears to have raised some challenges in the scientific community, particularly among allergologists and pneumologists, with regards to the optimal treatment choice both for individual patients and for the BA population overall. Indeed, the availability of mAbs directed towards IL-5 has led to questioning of the role of anti-IgE strategies in BA, regardless of disease phenotype (allergic vs non-allergic). The question is not whether an anti-IgE or an anti-eosinophilic strategy is more effective, but rather what the mechanism is at the origin of the airway inflammatory process or, in other words, what is the cause of the problem? In severe allergic asthma (SAA), both IgE and eosinophils are participants in a complex process in which they play different roles. It could be considered that IgE is the cause of allergic asthma, while eosinophilia is a consequence of the whole process. As a result of the ability of IgE antibodies to influence the functioning of several immune and structural cells of the bronchial wall, IgE is primarily responsible not only for the acute phase but also for the chronic phase of inflammation characteristic of BA, at least in allergic forms (Fig. 1a). This feature makes IgE an ideal target in the treatment of asthma [10]. In contrast, eosinophils, which are responsible for bronchial wall damage, are the final effector cells in the process (Fig. 1b). It is generally believed that in types of asthma with high eosinophilia (frequently non-allergic types), an anti-IL-5 strategy could be considered as the first choice of treatment [67–69]. Although the efficacy of mAbs towards IL-5 is indisputable in eosinophilic asthma, as reported in several studies, the clinical results are more evident in those patients with a high percentage of blood and sputum eosinophilia [58]. However, in clinical practice, the frequency and severity of asthma symptoms may not always be associated with eosinophil count, particularly in patients with blood eosinophilia close to the cut-off point identified as the predicting marker for prescribing an anti-IL-5 strategy. Of note, similar values of blood eosinophilia are predictors of response to treatment with both anti-IgE and anti-IL-5 mAbs [70, 71]. It is also important to be aware than omalizumab can reduce not only both blood and sputum eosinophils but also tissue eosinophil levels in bronchial biopsies [72].

Among specialists there is a common misconception that asthma is always associated with eosinophilic inflammation at the bronchial level. However, not all types of asthma are characterised by the eosinophilic pattern, there are also neutrophilic and pauci-granulocytic variants [73]. One concept that needs to be considered is that asthma endotypes can change over time, particularly regards the pattern of cellular components. One example may be represented by the viral-induced modification of the airway inflammatory responses, due to the release of pro-inflammatory cytokines and chemokines with may favour a Th1/Th17 immune response, or on the other hand, amplify a pre-existing Th2 response [74]. Although respiratory viral infections cause asthma exacerbations, data about the type of cellular infiltrate and underlying molecular mechanisms are not conclusive. The airway inflammatory responses during virus-induced exacerbations depend on the viral species and on host-related factors and may be associated with increases in neutrophils, eosinophils and macrophages [75–78].

From a clinical perspective, it is important to note that the efficacy of biological therapies is evident only in patients who have experienced asthma exacerbations. As previously mentioned, the question is not whether an anti-IgE or anti-eosinophil therapy is more effective but rather what is the cause and pathogenic mechanism prevalent in each patient. The complex biological role of IgE, how IgE mAbs achieve their clinical effects and the ability of IgE to regulate the functioning of several cells has been increasingly studied over the past few years by retracing our steps, that is, starting with the clinical observations of the effectiveness of omalizumab in severe asthma patients and then analysing how IgE mAbs achieved this effect. Indeed, unlike other biological agents, the clinical efficacy of omalizumab has been appreciated before fully understanding its overall mechanisms of action.

The discussion on the pleiotropic role of IgE appears more intriguing when taking into account the fact that omalizumab also exerts its therapeutic effects in non-allergic forms of asthma as well as in patients with nasal polyps [79, 80] This raises the question of how can these clinical findings be explained? Indeed, these data appear to contradict the well-established concept about the role of IgE only in allergic asthma. Two hypotheses have been proposed; the first assumes that in intrinsic asthma patients there is a local allergy, whereas the second hypothesis assumes that there is an immunological imbalance of DC function [81]. The role of IgE is suggested by the fact that in allergic and non-allergic asthma patients there is an increase of total and specific IgE levels in the serum, and an inverse relationship between IgE levels and lung function (FEV_1_) [82, 83]. Concerning local IgE production, some studies have shown dust mite-specific IgE in the bronchial secretions of intrinsic asthmatics as well as the production of IgE specific to *S. aureus* enterotoxin [82, 84].

Together, these findings suggest that IgE is the cause of allergic airway inflammation rather than the consequence of this process and that IgE could also play a role in intrinsic asthma. However, despite these biological and clinical data, scepticism still remains, and further studies in non-allergic asthmatic patients as well as dedicated clinical trials must be performed. In fact, while IgE is clearly upstream in allergic asthma, whether it remains the case in non-allergic asthma is far from being clear, and the role of eosinophils might become stronger.

### Targeting single vs multiple cytokines

While animal models have shown that antibody-mediated neutralisation of Th2 cytokines greatly diminishes airway inflammation [85–87], clinical trials of some biological agents have not always been particularly successful with positive trials frequently requiring patient stratification [88]. What has become clear is that the formation of cytokine–anti-cytokine immune complexes does not guarantee cytokine neutralisation; indeed, the formation of these complexes can, in some cases, potentiate target cytokine activity rather than neutralise it. For example, in a lebrikizumab (an anti-IL-13 mAb) dose-finding study in moderate-to-severe asthma patients with high periostin levels, there was a direct correlation between exacerbation rate and lebrikizumab dose [89]. Moreover, treatment with neutralising antibodies may also increase cytokine levels in the circulation; for example, mepolizumab-treated patients had higher circulating levels of IL-5 with the majority of it bound in IL-5–anti-IL-5 complexes [90], and treatment of asthma patients with anti-IL-13 antibodies directly increased serum levels of IL-13 [91]. These findings would suggest that direct targeting of the cytokine itself might not be particularly effective and that targeting a pathway or multiple cytokines might have a greater clinical effect.

## Biological agents targeting airway inflammation in asthma: The rational choice

Anti-IgE mAbs have been successfully used for more than 10 years in the treatment of BA, and now the spectrum of available biological drugs is expanding with different mAbs targeting different pathways involved in the pathogenesis of airways inflammation. Most cases of BA are related to an IgE-mediated pathogenic mechanism, at least in patients sensitised to allergens. Taking into account that IgE is clearly involved both at the onset of allergic asthma as well as during the chronic phase of the disease, it is perhaps unsurprising that omalizumab has proved to be clinically effective in SAA. The ability of omalizumab to reduce sputum eosinophilia and increase of the dose of methacholine required to induce a fall in FEV_1_ shows that in addition to inhibiting free IgE, omalizumab also has an effect on inflammatory cells [22]. It is well known that omalizumab selectively binds to free IgE molecules, blocking the binding site for both FcεRI and CD23 receptors, modulating and acting upstream of the IgE network and slowing or preventing the allergic inflammatory cascade. Although omalizumab does not have a direct effect on FcεRI, the depletion of free IgE induces a down-regulation of FcεRI expression, which interferes with the functioning of several FcεRI+ cells [92]. In this regard, omalizumab can down-regulate FcεRI on both myeloid and plasmocytoid DCs and, as a consequence, reduce the allergen-specific proliferative response of T cells. Not only do the T cells not proliferate, they do not produce cytokines (especially IL-3, IL-5, GM-CSF, and IL-13) actively involved in allergic inflammation [29]. So, through its effect on T cells, omalizumab exerts an indirect effect on eosinophils. Furthermore, it induces apoptosis of these cells, but does not result in lysis [72].

As a result of omalizumab exerting its therapeutic anti-inflammatory effects through several pathways, long-term treatment is associated with beneficial effects on airway remodeling by reversing already established histological changes [93]. As mentioned previously, several new biological agents have recently been launched that target specific pathways, particularly in patients with type-2-high or eosinophilic asthma, and are currently included in the therapeutic guidelines. Among these drugs, there is a great deal of interest in the anti-IL-5 mAbs mepolizumab and reslizumab, and the anti-IL-5R benralizumab. The importance of targeting IL-5 derives from its role in the induction of differentiation, survival and activation of eosinophils, which are key cells in a subgroup of BA.

The effect of mepolizumab and reslizumab is related to their ability to bind with high affinity to IL-5 and block the interaction between IL-5 and its receptor on the surface of eosinophils. The availability of anti-IL-5 mAbs has directed attention towards eosinophils as the main target of asthma treatment, neglecting the central role of IgE in SAA despite increasing awareness of the role of IgE in allergic inflammation. While among clinicians the importance of anti-IL-5 mAbs has been related to their ability to indirectly target eosinophils, some concerns have been raised. Anti-IL-5 mAbs have been unable to completely abolish both blood and airway eosinophils in asthma patients [94]; these data could be explained by cytokines others than IL-5, such as IL-3, GM-CSF, and IL-9, sustaining the eosinophilic airway inflammation [95, 96]. Furthermore, immunological research has clearly demonstrated that the cytokine network is a ‘redundant’ system in which several cytokines exert the same effects and one receptor can bind different cytokines.

Benralizumab, a humanised afucosylated mAb that binds the α-subunit of the IL-5 receptor, has a different mechanism of action for inhibiting the proliferation and activation of eosinophils, as well as efficiently depleting eosinophils by inducing apoptosis through antibody-dependent cell-mediated cytotoxicity [97]. Benralizumab is approved the US and under review in Europe and has been shown to be effective and safe as add-on therapy in patients with severe asthma and eosinophilia who are inadequately controlled with high-dose ICS plus LABA [98].

It is well known that a complex network of cytokines plays a role in the pathogenesis of BA. Among this network, attention has been given to IL-4 and IL-13. Indeed, a therapeutic strategy is based on the use of drugs able to interfere with the IL-4 and IL-13 pathways. IL-4 and IL-13 are involved in the production of IgE and the pathogenesis of several aspects of bronchial inflammation in asthma [99]. Dupilumab is a fully human mAb to the IL-4 receptor alpha (IL-4Ra) subunit that is activated by both IL-4 and IL-13 [100]. IL-4 is an essential factor in the differentiation of Th2 cells; both IL-4 and IL-13 induce the switch to IgE production; IL-13 induces the expression of adhesion molecules and is responsible for some of the changes seen in BA such as airway remodelling and smooth muscle hypertrophy. Dupilumab has demonstrated significant results in both eosinophilic and non-eosinophilic asthma, and in patients with atopic dermatitis [101].

Future directions in asthma treatment also include the use small molecules such as fevipiprant, an antagonist of the DP2 receptor, which has been shown to reduce eosinophilic airway inflammation in patients with moderate-to-severe asthma [102]. This receptor has been found to be expressed on major human cell types involved in asthma pathogenesis, such as Th2 lymphocytes, innate lymphoid cells (ILC) 2, eosinophils, and basophils [103].

## Conclusion

All the phases leading to inflammation of the airways, and therefore clinical expression of allergic asthma, must be considered as dynamic processes. Treatment decisions should consider the biological background that is “driving” the inflammation as this is likely to predict patients’ responses to treatment. While IgE is involved early in the inflammatory cascade and can be considered as a cause of allergic asthma, eosinophilia can be considered a consequence of the whole process. This helps to make sense of the clinical effectiveness observed with anti-IgE mAbs in bronchial asthma and the effects of anti-IL-5 mAbs in specific (eosinophilic) asthma patient populations.

However, the question of whether an anti-IgE or anti-eosinophil therapy will be more effective in the clinical practice with patients suffering from eosinophilic asthma is still an open question and further studies are needed.

## Abbreviations

ASMC: Airway smooth muscle cell
BA: Bronchial asthma
DC: Dentritic cell
FEV_1_: Forced expiratory volume in 1 s
GINA: Global Initiative for Asthma
GM-CSF: Granulocyte-macrophage colony stimulating factor
ICS: Inhaled corticosteroid
IFN: Interferon
IgE: Immunoglobulin E
IL: Interleukin
ILC2: Innate lymphoid cell 2
LABA: Long-acting β_2_-agonist
mAb: monoclonal antibody
PGD_2_: Prostaglandin D_2_
SAA: Severe allergic asthma
Th2 cell: T helper 2 cell
TNF-α: tumour necrosis factor-α
TSLP: Thymic stromal lymphopoietin
